# Evaluation of inflammatory biomarkers and their association with anti-SARS-CoV-2 antibody titers in healthcare workers vaccinated with BNT162B2

**DOI:** 10.3389/fimmu.2024.1447317

**Published:** 2024-08-23

**Authors:** Ester Leno-Duran, Esther Serrano-Conde, Ana Salas-Rodríguez, Inmaculada Salcedo-Bellido, Rocío Barrios-Rodríguez, Ana Fuentes, Laura Viñuela, Federico García, Pilar Requena

**Affiliations:** ^1^ Universidad de Granada, Departamento de Obstetricia y Ginecología, Granada, Spain; ^2^ Servicio de Microbiología, Hospital Universitario Clínico San Cecilio, Granada, Spain; ^3^ Universidad de Granada, Departamento de Medicina Preventiva y Salud Pública, Granada, Spain; ^4^ Instituto de Investigación Biosanitaria de Granada (ibs.GRANADA), Granada, Spain; ^5^ Centro de Investigación Biomédica en Red de Epidemiología y Salud Pública (CIBERESP), Madrid, Spain; ^6^ Centro de Investigación Biomédica en Red de Enfermedades Infecciosas (CIBERINFEC), Madrid, Spain

**Keywords:** COVID-19, BTN162b2, SARS-CoV-2, anti-spike antibodies, inflammatory biomarker

## Abstract

**Introduction:**

Vaccine-induced immunity against COVID-19 generates antibody and lymphocyte responses. However, variability in antibody titers has been observed after vaccination, and the determinants of a better response should be studied. The main objective of this investigation was to analyze the inflammatory biomarker response induced in healthcare workers vaccinated with BNT162b2, and its association with anti-Spike (a SARS-CoV-2 antigen) antibodies measured throughout a 1-year follow-up.

**Methods:**

Anti-spike antibodies and 92 biomarkers were analyzed in serum, along with socio-demographic and clinical variables collected by interview or exploration.

**Results:**

In our study, four biomarkers (ADA, IL-17C, CCL25 and CD8α) increased their expression after the first vaccine dose; and 8 others (uPA, IL-18R1, EN-RAGE, CASP-8, MCP-2, TNFβ, CD5 and CXCL10) decreased their expression. Age, body mass index (BMI), smoking, alcohol consumption, and prevalent diseases were associated with some of these biomarkers. Furthermore, higher baseline levels of T-cell surface glycoprotein CD6 and hepatocyte growth factor (HGF) were associated with lower mean antibody titers at follow-up, while levels of monocyte chemotactic protein 2 (MCP-2) had a positive association with antibody levels. Age and BMI were positively related to baseline levels of MCP-2 (β=0.02, 95%CI 0.00-0.04, p=0.036) and HGF (β=0.03, 95%CI 0.00-0.06, p=0.039), respectively.

**Conclusion:**

Our findings indicate that primary BNT162b2 vaccination had a positive effect on the levels of several biomarkers related to T cell function, and a negative one on some others related to cancer or inflammatory processes. In addition, a higher level of MCP-2 and lower levels of HGF and CD6 were found to be associated with higher anti-Spike antibody titer following vaccination.

## 1 Introduction

The coronavirus disease-2019 (COVID-19), an infectious respiratory disease caused by the SARS-CoV-2 virus, continues to place a global burden on morbidity and mortality. By spring 2024, there have been more than 770 millions COVID-19 cases and more than 7 millions deaths worldwide (1).

Several vaccines were developed throughout the year 2020 to provide protection against COVID-19. For the first time, vaccines based on mRNA were used in the general population. These vaccines, i.e. RNA-1273 (Spikevax™, Moderna) and BNT162b2 (Comirnaty^®^, Pfizer-BioNTech), contain mRNA encoding the Spike (S) protein of SARS-CoV-2 and in clinical trials provided around 95% protection against COVID-19 when two doses were administered (2, 3). They induce a humoral response with high levels of anti-S antibodies that decline after 3 or 6 months (4–6). Besides, the so-called cellular immunity mediated by T cells seems to play a relevant role in disease and vaccine-induced protection, what might be relevant in patients who do not have an adequate humoral response (7–10). Indeed, COVID-19 patients with poor antibody response like those receiving anti-CD20 therapy or cancer patients still induce strong CD4+ and CD8+ T cell responses (8, 9). CD4+T cells display several functions, including regulating the activity of B cells. They also play a role in activating CD8+ cytotoxic T cells, which are responsible for clearing intracellular viral infection.

Despite the effective immune response to BNT162b2 vaccination (11, 12), variability in antibody titers following vaccination has been observed by our group and other laboratories (13–17). Furthermore, some individuals do not develop a humoral response to natural SARS-CoV-2 infection (18, 19), which not necessarily imply a lack of immunity, as T-cells response could provide protection even in the absence of antibodies. Likewise, BNT162b2 vaccination induces a robust response of CD8+ and CD4+T cells that can vary among individuals (10). However, little is known about the immune mediators that are associated with a poorer response to the vaccine.

Understanding the factors that influence vaccine immune response may have important implications for enhancing the immunogenicity of SARS-CoV-2 vaccines. To achieve this goal, we analyzed the effect of BNT16b2 vaccination and sociodemographic and clinical variables on the levels of 92 inflammatory biomarkers in healthcare workers and the association of these biomarkers with anti-S antibodies.

## 2 Materials and methods

### 2.1 Study design and participants

An ambispective internal comparison cohort study was carried out. The study was conducted at Hospital Universitario Clínico San Cecilio (HUCSC), in the city of Granada (southern Spain) with a workforce of over 3000 employees. This research is part of a larger project whose main objective was to analyse the neutralizing capacity of antibodies after vaccination with different variants (13). The reference population consisted of healthcare and social-healthcare staff aged less than 65 years vaccinated with BNT162b2 (Cominarty^®^), as this was the only vaccine dispensed at the hospital for the first dose. After receiving the approval from the Ethic Committee in January 2021, participants were recruited in order of arrival to the vaccination event with no selection criteria restriction, and followed up for a year. A total of 147 participants were finally enrolled and all of them signed the informed consent to participate in the study. In a second part of this project and coinciding with the third vaccine dose administration in December 2021, participants were invited to continue the follow-up for an additional year. Besides, a questionnaire was administered at this timepoint, and permission was requested to analyse biomarkers in the blood samples previously donated. A total of 108 individuals provided their consent to participate in this second part.

### 2.2 Study samples and data collection

Serum samples were collected at the following times: just before/after the administration of the first dose of the vaccine (t0) and the second dose (t1). The time interval for sample collection was between few minutes before vaccination until a few hours later, being logistics the only reason for not being able to sample before. No record was taken on which participants sampled before or after the vaccination. Samples were also obtained 5 weeks after vaccination (t2), 3 months (t3), 8-9 months (t4) and 11 months (t5) after the first dose administration, the latter coinciding approximately with the administration of the third dose of the vaccine.

Sociodemographic (age, sex, educational level), lifestyle (smoking habit, alcohol consumption, Mediterranean diet adherence), and clinical information (chronic diseases) concerning the time of first vaccine dose administration was collected retrospectively using a self-administered questionnaire at t5 (**Supplementary Data Sheet**). Anthropometric data (body mass index and waist-hip ratio) was determined at the same visit by a dietician-nutritionist, and participants were asked whether they had changed weight since the first dose.

### 2.3 Determination of antibodies

Serum IgG antibodies were analyzed in the samples taken at the time points t0, t1, t2, t3, t4 and t5 using COVID-19 VIRCLIA IgG MONOTEST (VIRCELL, S.L., Spain) following manufacturer’s instructions. The assay is a qualitative indirect chemiluminescent immunoassay (CLIA) to test IgG antibodies in SARS-CoV-2 spike (S) and nucleocapsid (N), with a manufacturer declared 98% sensitivity and 99% specificity.

### 2.4 Determination of biomarkers

The analysis of 92 inflammatory biomarkers was performed on samples taken at the time points t0, t1 and t2. The list with full names and abbreviations of these markers is detailed in **Supplementary Table 1**. The Proximity Extension Assay technology, manufactured by Olink (Stockholm, Sweden) and performed at Cobiomic Bioscience, (Córdoba, Spain), was used. Briefly, this multiplex technique allows protein assays to be performed in a minimal clinical sample volume, using two matching antibodies labelled with unique DNA barcodes, for each target antigen. The protein in the solution that binds to both antibodies with sufficient stability is subsequently quantified by real-time PCR, providing higher sensitivity than classical immunoassays. The results obtained represent a relative value of protein expression compared to the buffer used for the PCR reaction. For the biomarkers IL-1α, IL-2, IL-4, IL-5, IL-13, IL-24, IL-33, IL-22RA1, beta-NGF and TSLP more than 75% of the samples had a value below the limit of detection and therefore were excluded from further analyses.

### 2.5 Statistical analysis

The Skewness-Kurtosis test was used to determinate the normal distribution of biomarker levels. Since most of the biomarkers did not exhibit parametric behaviour, the Friedman test was used to compare the levels of the biomarkers among the three timepoints after vaccination (t0, t1 and t2). Considering the large number of biomarkers analysed, a p-value correction for multiple comparisons was performed by means of the Benjamini-Hochberg method and q-values were estimated. As this correction may increase the probability of false type II errors, we also explored the borderline non-significant (q<0.1) results. In addition, a comparison between pairs of groups, namely t1 versus t0, and t2 versus t0, was performed using the Wilcoxon test. Then generalized estimating equations (GEE) were performed to test the association between baseline socio-demographic, lifestyle, anthropometric, and clinical variables (independent variables), and the change of inflammatory biomarker levels over time after vaccination (dependent variable). Of note, for age, BMI and hip-waist ratio, the change in cytokine expression refers to 1-unit increase.

To study the association between baseline biomarkers and antibody levels, the dimensionality of the biomarkers was reduced using a principal component analysis (PCA). It was not possible to perform a single PCA due to the small sample size and a large number of biomarkers. Therefore, it was decided to subdivide the biomarkers into groups according to their biological function (https://insight.olink.com/pathway-browser). The 4 biological pathways mostly covered by the biomarkers in the study were: 1) immune system (51 biomarkers); 2) signal transduction (42 biomarkers); 3) disease (14 biomarkers); and 4) protein metabolism and expression (12 biomarkers) (**Supplementary Table 2**). In addition, a fifth group was created under the name “Others’’ which included the 9 biomarkers not present in any of the above pathways. Biomarker repetition was allowed in the first 4 clusters. A PCA with no matrix rotation was performed for each of these 5 groups and the Kaiser–Meyer–Olkin (KMO) test, aimed to determine how suited the data are for PCA, was performed. Then, GEE were performed using the two principal components (PC1 and PC2) that contributed the most in the 5 PCAs done as the independent variables, and the repeated measures (t1, t2, t3, t4 and t5) of antibodies, as the dependent variable. For t0, t1 and t2, the samples were collected at practically the same time for all individuals, but for the rest of the timepoints there was variability, and the group mean was assigned to each participant (**Supplementary Table 3**). The analysis was adjusted for sex and age, as these two variables are well known to modify the immune system (20–22). In addition, the 5 individual biomarkers that contributed most to the PCs with a significant association with antibody levels were selected to evaluate their individual association with antibodies by means of GEEs. Furthermore, to evaluate the association of baseline socio-demographic, lifestyle, anthropometric, and clinical variables (independent variables), and selected baseline inflammatory biomarker levels (dependent variable), linear regression models were estimated.

Statistical significance was stated as p-value or q-value<0.05. Statistical analyses were performed using SPSS (version 28.0.1) and Stata SE 17.0 (Stata Corp, College Station, Texas) statistical softwares and graphs were constructed using GraphPad Prism 9.

## 3 Results

### 3.1 Description of the study cohort

The sample consisted of a total of 99 healthcare workers from the HUCSC with available data of both anti-S antibodies and inflammatory biomarkers. The characteristics of this cohort are provided in **Table 1**. Most part of the participants were female (78.8%) and the mean age was 47.1 years (standard deviation, SD: 11.9). 22.2% of the cohort had some chronic diseases. With regard to lifestyle, the majority were non-smoker (62.1%), and 49,4% did not drink alcohol. The adherence to the Mediterranean diet was high (mean=8.9, SD:1,7). Only 5 participants with available anti-S IgG data reported a SARS-CoV-2 positive diagnostic test between 3 and 10 months previous to vaccination. Antibody titers were similar to those with no previous infection (data not shown).

**Table 1:** Study population characteristics.

Sex (% female)	78.8
Age (years) mean (SD)	47.1 (11.9)
Body mass index (kg/m2) mean (SD)	25.3 (4.3)
Waist-hip ratio (cm) mean (SD)	0.8 (0.1)
Chronic disease (% yes)	22.2
Education level (%)	
Primary studies	20.7
Secondary studies	57.5
Higher	21.8
Smoking habit (%)	
Non-smoker	62.1
Former smoker	26.4
Smoker	11.5
Alcohol consumption (%)	
Nothing	49.4
Little	43.7
Much	6.9
MedD adherence score mean (SD)	8.9 (1.7)

### 3.2 Effect of vaccination on biomarker levels

**Figure 1:**
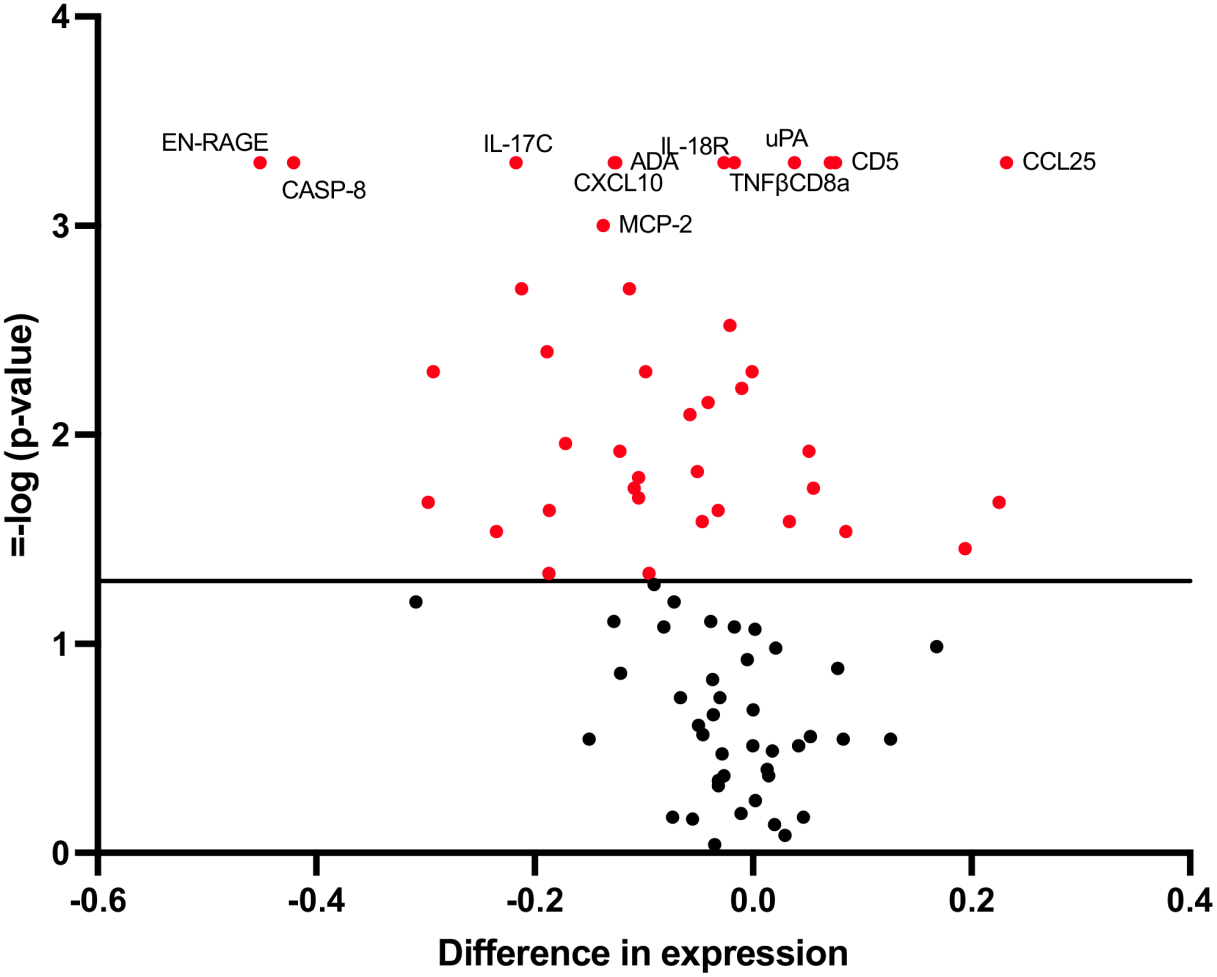
Changes in biomarker levels 5 weeks after vaccine administration. Volcano plots showing changes in biomarker levels comparing baseline versus 5 weeks after vaccination. Each circle represents one biomarker. The difference in expression is represented on the x-axis. The y-axis shows the log10 of the p value of the Friedman test, comparing baseline, 2-week and 5-week timepoints. A p value<0.05 is indicated by a red dot.

Of the 82 biomarkers finally included in the analysis, the expression of 41 was modified after the vaccination process showing a p-value<0.05 (**Figure 1**; **Supplementary Table 4**). However, after adjustment for multiple comparisons, only 11 biomarkers retained the statistical significance: CD8α, UPA, IL-17C, IL-18R1, CD5, CXCL10, EN-RAGE, CASP-8, CCL25, ADA and TNFβ. In addition, the biomarker MCP-2 had a q-value very close to 0.05 and was also considered for further analyses (**Figure 1**; **Supplementary Table 4**).

**Figure 2:**
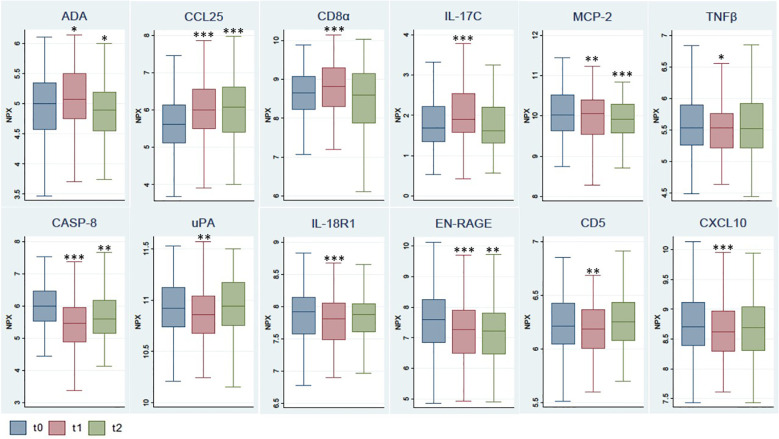
Comparison of selected biomarker levels at different timepoints after vaccination. The box shows the median, and 25th and 75th percentiles. Outliers have not been plotted in the graph. *p<0.05, **p<0.01, ***p<0.001 for Wilcoxon test, with reference t0. NPX, Normalized Protein eXpression.

The distribution of values for these 12 markers in each timepoint sample as well as the differences between groups are shown in **Figure 2**. A statistically significant increase was observed 3 weeks after the administration of the first dose of the vaccine (t1, coinciding with the administration of the second dose) in the biomarkers ADA, CCL25, CD8α and IL-17C. This significant increase was maintained 5 weeks later (t2) for CCL25, while a decrease was observed for ADA. On the other hand, a statistically significant decrease was observed 3 weeks after the administration of the first dose in the biomarkers MCP-2, CASP-8, TNFβ, uPA, IL-18R1, EN-RAGE, CD5 and CXCL10. This significant decrease was maintained over the following 5 weeks for MCP-2, CASP-8 and EN-RAGE.

### 3.3 Effect of socio-demographic and clinical variables on biomarkers affected by the vaccination

Next, we assessed which baseline socio-demographic and clinical variables may be associated with the mean levels over time of the 12 biomarkers modified after the vaccination process. Older age was significantly associated with a higher mean concentration of CCL25. On the other hand, smoking habit, consumption of high level of alcohol and the presence of any illness were related to a lower mean concentration of CCL25, CD8α and IL-17c, respectively (**Table 2**).

**Table 2:** Association of socio-demographic and clinical variables with biomarkers with increased levels after 3 weeks of vaccination.

Variables	ADA Mean difference (95%CI)	CCL25 Mean difference (95%CI)	CD8α Mean difference (95%CI)	IL-17c Mean difference (95%CI)
Age (years)	0.00 (-0.01, 0.01) p=0.968	**0.02 (0.01, 0.04)** p=0.002	-0.01 (-0.02, 0.00) p=0.106	0.00 (-0.01, 0.02) p=0.702
Sex	0.05 (-0.21, 0.30) p=0.726	0.14 (-0.27, 0.55) p=0.505	0.24 (-0.20, 0.69) p=0.285	-0.18 (-0.66, 0.31) p=0.473
BMI (kg/m2)	0.00 (-0.03, 0.03) p=0.953	-0.00 (-0.04, 0.03) p=0.790	-0.01 (-0.05, 0.03) p=0.707	0.01 (-0.03, 0.05) p=0.572
Waist-hip ratio (cm)	0.64 (-1.09, 2.37) p=0.469	-0.61 (-2.88, 1.67) p=0.601	-0.02 (-2.98, 2.94) p=0.989	1.33 (-1.25, 3.91) p=0.311
Adherence to MD	0.00 (-0.05, 0.06) p=0.816	0.05 (-0.01, 0.11) p=0.135	-0.05 (-0.11, 0.02) p=0.196	-0.00 (-0.06, 0.05) p=0.875
Presence of illness	0.07 (-0.13, 0.27) p=0.518	-0.08 (-0.42, 0.26) p=0.642	-0.12 (-0.51, 0.27) p=0.550	**-0.41 (-0.72, -0.10)** p=0.009
Alcohol consumption^1^ Low High	0.04 (-0.15, 0.22) p=0.676 0.25 (-0.02, 0.52) p=0.070	-0.04 (-0.33, 0.24) p=0.773 -0.34 (-0.89, 0.21) p=0.231	-0.13 (-0.42, 0.15) p=0.356 **-0.66 (-1.34, 0.02)** p=0.056	0.01 (-0.26, 0.27) p=0.959 -0.25 (-0.74, 0.24) p=0.310
Smoking status^1^ Former smoker Smoker	-0.00 (-0.23, 0.22) p=0.979 -0.11 (0.36, 0.14) p=0.374	0.01 (-0.31, 0.33) p=0.963 **-0.50 (-0.82, -0.18)** p=0.002	-0.01 (-0.38, 0.37) p=0.972 -0.14 (-0.52, 0.25) p=0.484	0.02 (-0.31, 0.36) p=0.892 -0.12 (-0.47, 0.24) p=0.522
Level education^2^ Secondary Higher	-0.05 (-0.26, 0.16) p=0.649 -0.14 (-0.40, 0.11) p=0.271	-0.01 (-0.59, 0.25) p=0.966 -0.17 (-0.59, 0.25) p=0.434	-0.24 (-0.61, 0.14) p=0.214 -0.01 (-0.45, 0.43) p=0.968	0.17 (-0.14, 0.48) p=0.292 0.29 (-0.13, 0.71) p=0.177

Cells provide the mean difference over time (plus de 95%CI -confidence interval) in cytokine levels per unit-increase (continue variables) or per category change (categoric variables). p-value correspond to the generalized estimating equation models. BMI, body mass index; MD, mediterranean diet; ^1^Never as reference category; ^2^None or primary as reference category.

Statistically significant (<0.05) p-values are marked in bold.

With regards to the biomarkers with decreased changes over time after 3 weeks of vaccination, age was related with increased mean of MCP-2 and CXCL10, and with a decreased mean of IL-18Rl. Higher BMI and waist-hip ratio were associated with IL-18Rl and CD5, respectively. High alcohol consumption was related with a mean increase in MCP-2. Besides, CASP-8 and EN-RANGE had a positive association with the smoking habit and the level of education (**Table 3**).

**Table 3:** Association of socio-demographic and clinical variables with decreased biomarkers levels after vaccination.

Variables	MCP-2 Mean difference (95%CI)	TNFβ Mean difference (95%CI)	CASP-8 Mean difference (95%CI)	UpA Mean difference (95%CI)	IL18R1 Mean difference (95%CI)	EN-RANGE Mean difference (95%CI)	CD5 Mean difference (95%CI)	CXCL10 β (95%CI)
Age	**0.02 (0.00, 0.03)** p=0.019	-0.00 (-0.01, 0.01) p=0.497	0.00 (-0.01, 0.02) p=0.495	0.00 (-0.00, 0.01) p=0.174	**-0.00 (-0.02, -0.00)** p=0.047	-0.00 (-0.02, 0.02) p=0.951	-0.00 (-0.01, 0.00) p=0.653	**0.01 (0.00, 0.02)** p=0.023
Sex	0.15 (-0.37, 0.68) p=0.566	-0.20 (-0.49, 0.09) p=0.174	-0.04 (-0.42, 0.32) p=0.810	-0.05 (-0.22, 0.13) p=0.615	-0.15 (-0.40, 0.09) p=0.219	-0.02 (-0.58, 0.55) p=0.957	-0.14 (-0.35, 0.06) p=0.163	0.11 (-0.36, 0.58) p=0.649
BMI	0.02 (-0.02, 0.05) p=0.287	0.01 (-0.01, 0.03) p=0.367	0.01 (-0.02, 0.05) p=0.484	0.00 (-0.01, 0.02) p=0.767	**0.03 (0.01, 0.06)** p=0.010	0.0 (-0.05, 0.05) p=0.915	-0.01 (-0.02, 0.01) p=0.489	0.02 (-0.01, 0.06) p=0.127
Waist-hip ratio	1.05 (-2.35, 4.46) p=0.543	0.99 (-0.76, 2.75) p=0.269	1.01 (-1.14, 3.18) p=0.356	0.09 (-0.78, 0.96) p=0.836	1.42 (-0.14, 2.97) p=0.074	-0.18 (-3.51, 3.15) p=0.914	**1.10 (0.04, 2.15)** p=0.042	1.07 (-0.94, 3.07) p=0.299
Adherence to MD	-0.03 (-0.13, 0.07) p=0.561	-0.03 (-0.08, 0.01) p=0.185	-0.01 (-0.08, 0.06) p=0.818	0.01 (-0.01, 0.04) p=0.272	0.01 (-0.03, 0.05) p=0.799	0.04 (-0.05, 0.13) p=0.345	-0.01 (-0.04, 0.02) p=0.416	0.01 (-0.05, 0.08) p=0.702
Illness	-0.20 (-0.69, 0.28) p=0.413	-0.14 (-0.35, 0.08) p=0.216	-0.01 (-0.30, 0.28) p=0.943	-0.02 (-0.16, 0.11) p=0.694	-0.10 (0.32, 0.11) p=0.356	0.18 (-0.31, 0.67) p=0.471	-0.06 (-0.24, 0.11) p=0.479	-0.01 (-0.28, 0.26) p=0.940
Alcohol^1^ Low High	-0.02 (-0.32, 0.27) p=0.871 **0.57 (0.22, 0.93)** p=0.002	0.11 (-0.10, 0.31) p=0.305 -0.14 (-0.42, 0.14) p=0.319	-0.15 (-0.42, 0.11) p=0.253 -0.04 (-0.45, 0.37) p=0.843	0.03 (-0.08, 0.14) p=0.599 -0.09 (-0.27, 0.09) p=0.310	0.04 (-0.14, 0.22) p=0.668 0.17 (-0.11, 0.45) p=0.236	-0.28 (-0.69, 0.13) p=0.178 0.09 (-0.44, 0.63) p=0.736	0.02 (-0.10, 0.16) p=0.622 -0.05 (0.30, 0.20) p=0.706	0.27 (-0.01, 0.57) p=0.066 0.05 (-0.30, 0.40) p=0.789
Smoking status^1^ Former smoker Smoker	-0.09 (-0.47, 0.29) p=0.652 0.10 (-0.26, 0.45) p=0.592	-0.17 (-0.40, 0.07) p=0.161 0.21 (-0.02, 0.43) p=0.074	-0.23 (-0.59, 0.12) p=0.193 **0.54 (0.11, 0.97)** p=0.014	0.02 (-0.12, 0.17) p=0.761 0.13 (-0.01, 0.26) p=0.075	0.10 (-0.09, 0.29) p=0.315 -0.11 (-0.36, 0.13) p=0.353	-0.121 (-0.68, 0.44) p=0.673 **0.53 (0.04, 1.02)** p=0.036	0.01 (-0.13, 0.15) p=0.882 0.11 (-0.18, 0.39) p=0.466	0.11 (-0.20, 0.41) p=0.491 -0.18 (-0.55, 0.18) p=0.322
Level education^2^ Secondary Higher	-0.08 (-0.41, 0.25) p=0.631 0.31 (-0.12, 0.73) p=0.159	0.16 (-0.10, 0.42) p=0.234 0.24 (-0.01, 0.49) p=0.061	** 0.53 (0.19, 0.88)** p=0.003 0.36 (-0.03, 0.74) p=0.067	0.04 (-0.11, 0.18) p=0.623 -0.03 (-0.21, 0.15) p=0.728	-0.02 (-0.24, 0.20) p=0.840 -0.08 (-0.34, 0.19) p=0.571	** 0.70 (0.22, 1.17)** p=0.004 0.53 (-0.04, 1.10) p=0.070	-0.08 (-0.24, 0.07) p=0.290 -0.14 (-0.33, 0.06) p=0.166	-0.04 (-0.45, 0.36) p=0.819 -0.03 (-0.51, 0.45) p=0.899

Cells provide the mean difference over time (plus de 95%CI -confidence interval) in cytokine levels per unit-increase (continue variables) or per category change (categoric variables). p-value correspond to the generalized estimating equation models. BMI, body mass index; MD, mediterranean diet; ^1^Never as reference category; ^2^None or primary as reference category.

Statistically significant (<0.05) p-values are marked in bold.

### 3.4 Association of baseline biomarker levels with post-vaccination antibody levels

A PCA was performed for all 5 functional biomarker groups and KMO values above 0.6 were obtained in all cases (**Supplementary Tables 5**–**9**). An association of mean antibody levels over follow-up was observed with PC2-Others and PC1-Signal Transduction (**Table 4**).

**Table 4:** Association of PC1 and PC2 of different functional biomarker groups with the mean antibody levels at follow-up.

	Beta	95% CI	p-value
*PC1 Disease*	-87.4	-263.6; 88.6	0.330
*PC2 Disease*	-57.1	-282.6; 168.4	0.620
*PC1Protein Metabolism*	-108.6	-286.0; 68.9	0.230
*PC2 Protein Metabolism*	50.3	-191.3; 91.9	0.683
*PC1 Others*	-150.3	-314.7;14.0	0.073
***PC2 Others***	**297.7**	**30.2; 565.3**	**0.029**
*PC1 Immune system*	-84.2	-182.3; 13.9	0.092
*PC2 Immune system*	24.4	-130.7; 179.4	0.758
***PC1 Signal Transduction***	**-95.7**	**-184.8; -6.5**	**0.035**
*PC2 Signal Transduction*	27.9	-146.7; 202.4	0.754

CI, confidence interval. Statistically significant (<0.05) p-values are marked in bold.

**Figure 3:**
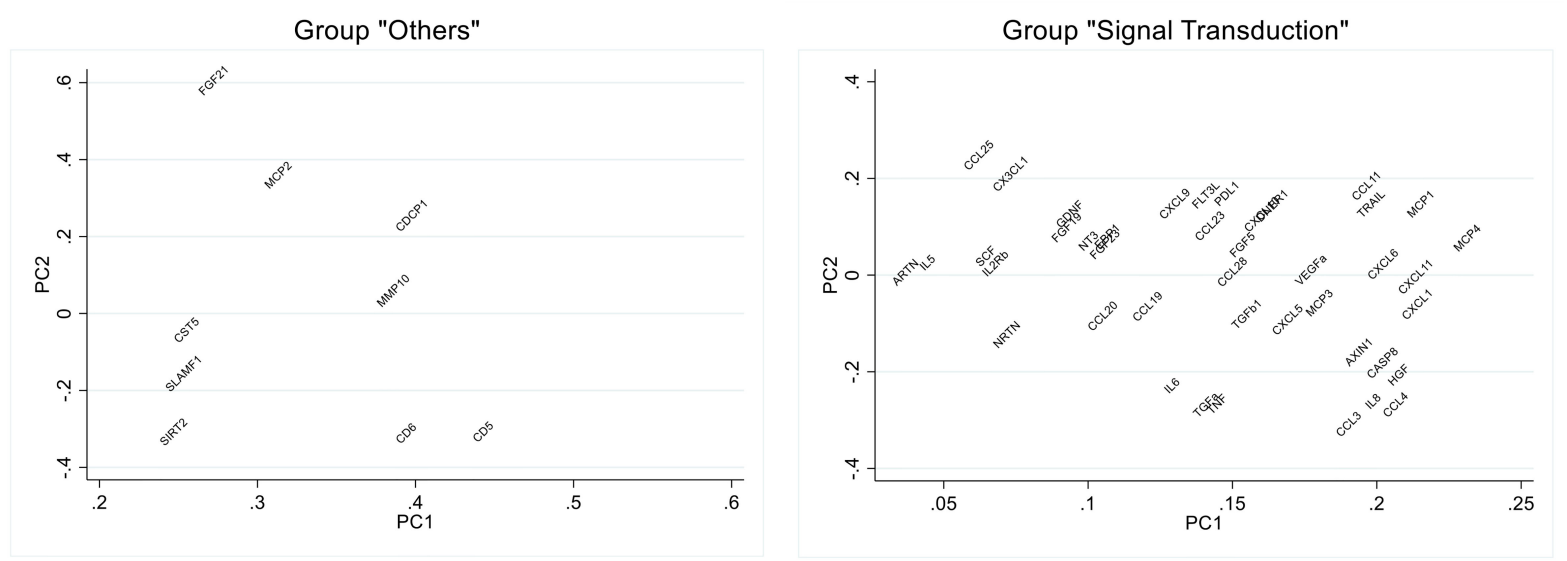
Loading plot of the principal component analysis of selected biomarker groups. Eigen vector values for the principal component (PC) 1 and PC2 are shown in the graph.

The biomarkers that contributed most to these PCs (**Figure 3**; **Supplementary Tables 5**, **6**) were: CD5, CD6, SIRT2, MCP-2 and FGF-21 (PC2 Others); HGF, CXCL1, CXCL11, MCP-1 and MCP-4 (PC1 Signal Transduction). In the analysis of their individual associations with antibody levels, 2 of the biomarkers (CD6 and HGF) showed a significant negative association, and the biomarker MCP-2 obtained a positive association very close to significance (**Table 5**).

**Table 5:** Association between baseline individual biomarkers and post-vaccination antibody levels at follow up.

	Beta	95% CI	p-value
CD5	-496	-1202.0; 210.0	0.169
**CD6**	**-603.2**	**-1045.6; -160.8**	**0.008**
CXCL1	-199.1	-740.2; 341.9	0.471
CXCL11	-52.9	-462.9; 357.0	0.800
FGF-21	6.6	-244.9; 258.1	0.959
**HGF**	**-745.8**	**-1508.4; 16.8**	**0.055**
MCP-1	-139.6	-680.3; 401.1	0.613
**MCP-2**	**382.7**	**-39.9; 805.3**	**0.076**
MCP-4	-313.9	-789.1; 161.4	0.196
SIRT2	-284.9	-685.6; 115.8	0.163

CI, confidence interval. Statistically significant (<0.05) or borderline (<0.1) p-values are marked in bold.

**Figure 4:**
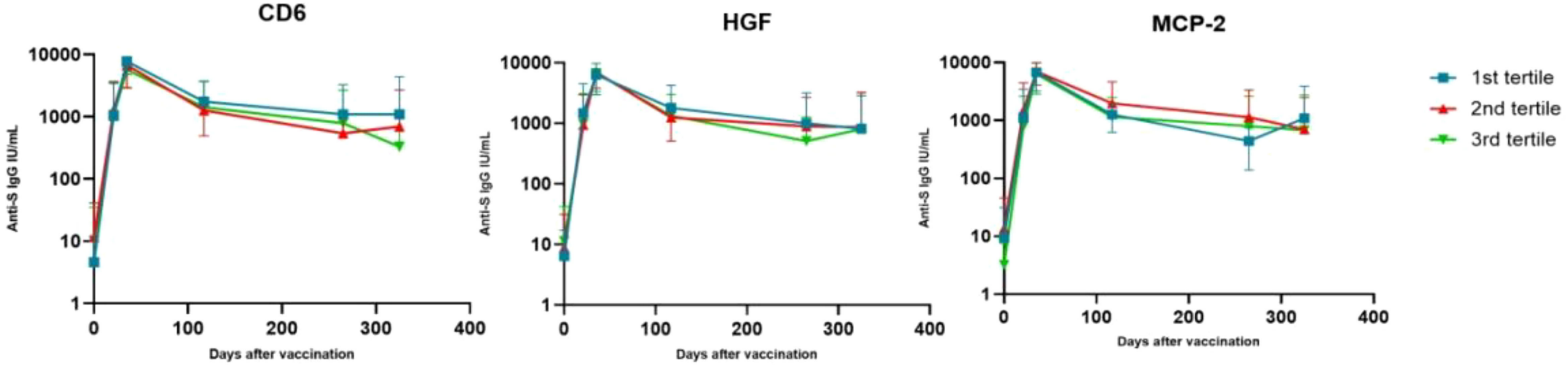
Changes in antibody levels after vaccine administration, stratifying individuals according to their levels of certain biomarkers. Values represent mean and standard deviation. On the x-axis represents the days after vaccination and on the y-axis the mean antibody concentration on a logarithmic scale in base 10.

In order to interpret the results more easily, we created a graph for each of these 3 biomarkers categorized by tertiles (**Figure 4**).

The highest antibody levels were similar for all individuals (at t2), regardless of their HGF, CD6 or MCP-2 levels. But the drop in antibodies at t4 was more pronounced for those individuals with higher levels (3rd tertile) of CD6 and HGF, and those individuals with lower levels (1st tertile) of MCP-2. This drop continued for CD6 at t5.

### 3.5 Effect of socio-demographic and clinical variables on baseline biomarkers related to the antibody responses after vaccination

Next, we assessed which socio-demographic and clinical variables may be associated with the levels of those biomarkers related to the antibody response, i.e. CD6, HGF and MCP-2. BMI was positively associated with HGF (β=0.03, 95% CI 0.00, 0.06, p=0.039) and age with MCP-2 (β=0.02, 95% CI 0.00, 0.04, p=0.036, respectively).

## 4 Discussion

The results show that our study cohort, which was followed up for approximately 1 year, presented changes in 12 inflammatory biomarkers measured after the BNT162b2 vaccination process; 4 of them showed increased expression (ADA, CD8α, IL-17C and CCL25), and 8 others (uPA, IL-18R1, EN-RAGE, CASP-8, MCP-2, TNFβ, CD5 and CXCL10) decreased.

All biomarkers whose expression increased after vaccination are related to T-lymphocyte function. Thus, ADA (adenosine deaminase) is essential for lymphocyte production and its deficiency is a cause of severe combined immunodeficiency (23). Consistent with our findings, upregulation of ADA has been detected after BNT16b2 vaccination (24, 25), as well as after SARS-CoV-2 infection (24). CD8 is a cell surface receptor on cytotoxic T cells, necessary for the recognition of other cells, and CD8+ T cells induced by vaccination may contribute to humoral and long-term immunity (26–29). IL-17C is a member of the IL-17 family that is selectively induced in the epithelium and stimulates Th17 lymphocyte activity (30). Finally, CCL25 is specifically expressed in the thymus and intestinal epithelium, and is involved in T-lymphocyte development (31, 32). Considering its localization in the mucosa, CCL25 may play an important role in attracting T cell and IgA antibody secreting cells to the mucosa (33, 34), and it has been proposed as a viable candidate for mucosal adjuvant in vaccines to boost systemic and mucosal immunity (35). Altogether, these observations highlight the relevance of the T-lymphocyte-mediated cellular response in BNT162b2 vaccine-induced immunity. Of note, unhealthy lifestyles and the presence of a prevalent disease were negatively associated with the expression of some of these biomarkers, suggesting that these conditions may also have a negative impact in the cellular immune response induced by the vaccination.

Some biomarkers whose expression was inhibited after vaccination are related to cancer. Thus, uPA is an urokinase involved in the processes of cancer invasion and metastasis that participates in the degradation of the extracellular matrix and maintains the cohesion of normal cells in tissues (36). Moreover, it modulates inflammation through release pro-inflammatory cytokines. The levels of uPA and its receptor uPAR in the lung have been related to severe outcomes of COVID-19 like the acute respiratory distress syndrome (37–39), lung injury and risk of mortality (40, 41). In addition, CASP-8 plays a key role in regulating cell apoptosis. In consonance with our results showing decreased expression of these biomarkers after vaccination, it has been proposed that one of the mechanisms by which the COVID-19 mRNA vaccine induces lung protection is through the inhibition of apoptosis of epithelial and endothelial cells (42). Another anti-apoptotic effect suggested is mediated PPAR agonists and the upregulation of anti-apoptotic factors such as MCL-1 (43). Other biomarkers inhibited in the weeks following administration of BNT162b2 are related to inflammatory processes, e.g. EN-RAGE, IL-18R1 and CXCL10 (44, 45). Of note, high levels of these biomarkers have been associated with risk of SARS-CoV-2 infection or COVID-19 severity (24, 39, 46–54), what suggests, in line with our results, that these biomarkers hamper the development of either natural or artificial immunity against COVID-19. And in agreement with our results, some studies have shown that CXCL10 levels decrease after COVID-19 vaccination (24, 51, 53, 54). As expected, the expression of some of these pro-inflammatory and pro-cancer markers were positively associated with unhealthy lifestyles or conditions (smoking habit, alcohol consumption or higher BMI).

With respect to biomarkers and their association with the post-vaccination humoral response, higher baseline levels of MCP-2, and, HGF and CD6 were associated with higher and lower mean antibody titers respectively at follow-up. MCP-2, also known as CCL8, is a chemokine produced by a wide variety of cells in response to other cytokines such as IL-1, IFN-gamma, etc. This chemokine, at high concentrations, behaves as a potent activator of eosinophils and basophils (55) and its main function is to attract monocytes, lymphocytes, and other types of inflammatory cells to the site of inflammation or injury. Other functions include cell proliferation and angiogenesis, as well as modulation of the inflammatory response by interacting with macrophages and dendritic cells. Thus, individuals with higher levels of MCP-2 may induce a greater influx of monocytes and lymphocytes to the site of vaccination, ultimately inducing a more durable antibody response. Indeed, a chemokine signature including higher plasma level of MCP-2 has previously been shown in COVID-19 patients compared to healthy controls (52, 56–58). In addition, our analysis showed that MCP-2 was positively associated with age. This would suggest that age may be associated with a stronger antibody response mediated by MCP-2. However, some of our unpublished data and others have shown precisely the opposite, that age is inversely associated with the antibody response after vaccination (59, 60). This controversy may be explained by other factors related to age, like thymic involution or comorbidities that may contribute more strongly to a poor antibody response than MCP-2 production.

HGF, also known as hepatocyte growth factor, is a signaling protein that plays an important role in tissue development regeneration and immune response. In addition, HGF has been found to promote cell survival, tissue protection and regeneration, but restricts fibrosis and inflammation (61). By inhibiting inflammation, HGF may have the opposite effect to MCP-2, not allowing the correct arrival of leukocytes at the site of vaccine administration, which would explain why individuals with higher levels of HGF would eventually experience a drop in antibody levels.

Finally, CD6 is a type I transmembrane glycoprotein, expressed almost entirely by lymphocytes. Its functions are still a matter of study, but some of them include co-stimulation of T cells, thereby enhancing a more effective immune response, or cell adhesion, facilitating interaction between immune cells (62). Given this biological function, it does not seem logical that individuals with the highest CD6 levels would have a worse antibody response at follow-up. However, it should be noted that the physiological effect described above occurs with the membrane marker but in our study, we measured the soluble (serum) biomarker. Further studies are needed to confirm the role of CD6 in the antibody response after vaccination.

This study investigated a large number of biological biomarkers in BNT162b2-vaccinated healthcare workers. The immune response induced by mRNA vaccination has been studied by several researchers (4, 63–65). Cellular and humoral immune memory responses, including T cell activity, have been detected in vaccinated individual (4). Kramer et al., identified a specific population of CD4^+^ CD8^+^ ICOS^+^ CD38^+^ CXCR5^-^ in response to BNT162b2 vaccine (63). Arunachalam et al., showed a signature of innate antiviral immunity with a higher level of plasma INFγ and CD14^+^CD16^+^ inflammatory monocytes (65). Moreover, another study have identified a platelet humoral response with change in proinflammatory cytokines (IL-1β, IFNγ, TNF-α between others) and anti-inflammatory cytokines (IL-10) (64).

Our study has some limitations. Firstly, the study sample is relatively small. In addition, this study was conducted in a single health center and all participants were health professionals, which may limit the representativeness of the population. Finally, there was some variability in the sample timing collection. For t0 and t1, samples were collected the same day but before or after the vaccination, while samples corresponding to t4 and t5 samples were obtained at different days after vaccination. This could potentially lead to a bias towards the null in the analysis. Yet, we did observe some interesting significant results which make our study relevant despite this limitation.

Among the strengths of our study, it is found the follow-up of participants, with six antibody measurements over a one-year period, which allowed us to characterize the post-vaccination immune response properly. Moreover, the large number of biomarkers studied allowed us to examine the expression of molecules that are not usually included in this type of study, and which provide very novel information.

## 5 Conclusions

In summary, the present study revealed that after primary BNT162b2 vaccination the biomarkers ADA, IL-17C, CCL25 and CD8α, which are related to T-cell function, were found to have increased expression. In contrast, the biomarkers uPA, IL-18R1, EN-RAGE, CASP-8, MCP-2, TNFβ, CD5 and CXCL10, whose functions are related to cancer or inflammatory processes, had their expression inhibited. In addition, higher levels of MCP-2 were associated with higher mean anti-S antibody titers after vaccination, whereas HGF and CD6 levels showed a negative association with mean antibody titers.

## Data Availability

The raw data supporting the conclusions of this article will be made available by the authors, without undue reservation.
