# Supplementary material for: Evaluation of inflammatory biomarkers and their association with anti-SARS-CoV-2 antibody titers in healthcare workers vaccinated with BNT162B2
Source: Front Immunol. 2024 Aug 23;15:1447317. doi: 10.3389/fimmu.2024.1447317 (PMC11377239; doi:10.3389/fimmu.2024.1447317)
Supplement: Supplementary file 1 [file DataSheet1.docx]

***Supplementary Information***

*Questionnaire*

| Interview date | | | | | Participant code (sticker) | | | | | | | | | | | | |
| --- | --- | --- | --- | --- | --- | --- | --- | --- | --- | --- | --- | --- | --- | --- | --- | --- | --- |
| Sex | | | | | Birth date | | | | | | | | | | | | |
| Service in which you work | | | | | Professional category | | | | | | | | | | | | |
| Height (meters) | | | | |  | | | | | | | | | | | | |
| Have you changed weight and if so how much, since you were given the first dose of the vaccine (in the last half year)? | | | | | | | | | | | | | | | | | |
| Mark with an X what is the highest level of education you have completed:  Primary / EGB Baccalaureate / BUP / COU Medium Degree / Technical specialist  Degree / Degree +4 years Diploma / Higher Degree  Master / Doctorate Other | | | | | | | | | | | | | | | | | |
| **DIET** | | | | | | | | | | | | | | | | | |
| **Answer the following questions according to your current eating and cooking habits (Third dose) and approximately 6 months ago (First dose)** | | | | | | | | | Third dose | | | | | First dose | | | |
| 1. Do you use olive oil as the main fat for cooking? | | | | | | | | | Yes | | | | No | Yes | | | No |
| 1. Do you consume 4 or more tablespoons of olive oil in total per day (including the one used for frying, toast, salads, etc.)? | | | | | | | | | Yes | | | | No | Yes | | | No |
| 1. Do you consume 2 or more servings of vegetables or vegetables per day? (the garnishes or accompaniments = 1/2 serving) | | | | | | | | | Yes | | | | No | Yes | | | No |
| 1. Do you consume 3 or more pieces of fruit (including natural juice) per day? | | | | | | | | | Yes | | | | No | Yes | | | No |
| 1. Do you consume less than 1 serving of red meats, hamburgers, sausages or sausages per day? (portion: 100 - 150 g) | | | | | | | | | Yes | | | | No | Yes | | | No |
| 1. Do you consume less than 1 serving of butter, margarine or cream per day? (individual portion: 12 g) | | | | | | | | | Yes | | | | No | Yes | | | No |
| 1. Do you consume less than 1 carbonated and / or sugary drink (sodas, colas, tonics, bitter) per day ? | | | | | | | | | Yes | | | | No | Yes | | | No |
| 1. Do you consume 7 or more glasses / glasses of wine per week? | | | | | | | | | Yes | | | | No | Yes | | | No |
| 1. Do you consume 3 or more servings of legumes per week? | | | | | | | | | Yes | | | | No | Yes | | | No |
| 1. Do you consume 3 or more servings of fish-seafood per week? (1 serving: 100 - 150 of fish or 4-5 pieces or 200 g of seafood) | | | | | | | | | Yes | | | | No | Yes | | | No |
| 1. Do you consume less than 2 times a week commercial pastry (not homemade) like cookies, flans, sweet or cakes a week? | | | | | | | | | Yes | | | | No | Yes | | | No |
| 1. Do you consume 3 or more times a week nuts? (portion 30g) | | | | | | | | | Yes | | | | No | Yes | | | No |
| 1. Do you prefer chicken, turkey or rabbit meat instead of beef, pork, hamburgers or sausages? (If vegetarian, mark Yes) | | | | | | | | | Yes | | | | No | Yes | | | No |
| 1. Do you consume 2 or more times a week the cooked vegetables, the pasta, rice or other dishes seasoned with tomato sauce, garlic, onion or leek made slowly with olive oil (sofrito)? | | | | | | | | | Yes | | | | No | Yes | | | No |
| **TOBACCO** | | | | | | | | | | | | | | | | | |
| In your life, have you smoked 100 or more cigarettes? | | | Yes | | | | No | | | | | | | | | | |
| Do you currently smoke? | | | Yes | | | | No | | | | | | | | | | |
| If you have been a smoker but no longer smoke, when did you quit smoking? | | | | | | | | | | | | | | | | | |
| If you are a smoker, how much do you smoke on average? | | | | | | | | | | | | | | | | | |
| How much did you smoke during the first dose of the vaccine? | | | | | | | | | | | | | | | | | |
| At what age did you start smoking regularly, more than 1 cigarette a week? | | | | | | | | | | | | | | | | | |
| **ALCOHOL** | | | | | | | | | | | | | | | | | |
| How many alcoholic drinks do you consume per week currently? | | | | | How many alcoholic drinks did you consume per week when you were given the first dose of the vaccine? | | | | | | | | | | | | |
| Never or almost never | | | | | Never or almost never | | | | | | | | | | | | |
| ≤7 spread throughout the week | | | | | ≤7 spread throughout the week | | | | | | | | | | | | |
| ≤7 concentrated in 1-2 days | | | | | ≤7 concentrated in 1-2 days | | | | | | | | | | | | |
| >7 spread throughout the week | | | | | >7 spread throughout the week | | | | | | | | | | | | |
| >7 concentrated in 1-2 days | | | | | >7 concentrated in 1-2 days | | | | | | | | | | | | |
| At what age did you start drinking with that regularity? | | | | | | | | | | | | | | | | | |
| Preferred type of drink: Beer Wine Spirits/Distilled | | | | | | | | | | | | | | | | | |
| **PHYSICAL ACTIVITY:** What physical activity have you done during the last month or in your usual month? | | | | | | | | | | | | | | | | | |
| Answer the following questions according to your physical activity habits at the current moment (Third Dose) and approximately 6 months ago (First Dose). | | | | | | | | | | | | | | | | | |
| **Walk** |  |  | |  | |  | | |  | | | | |  | | | |
| 3rd Dose |  | Minutes/day | |  | | Days/month | | | |  | | | |  | | | |
| 1st Dose |  | Minutes/day | |  | | Days/month | | | | |  | | |  | | | |
| **Work in the garden** |  |  | |  | |  | | | | |  | | |  | | | |
| 3rd Dose |  | Minutes/day | |  | | Days/month | | | | |  | | |  | | | |
| 1st Dose |  | Minutes/day | |  | | Days/month | | | | |  | | |  | | | |
| **Play sports or dance** |  |  | |  | |  | | | | |  | | |  | | | |
| 3rd Dose |  |  | |  | |  | | | | |  | | |  | | | |
| Type of sport |  | Minutes/day | |  | | Days/month | | | | |  | | |  | | | |
| Type of sport |  | Minutes/day | |  | | Days/month | | | | |  | | |  | | | |
| Type of sport |  | Minutes/day | |  | | Days/month | | | | |  | | |  | | | |
| 1st Dose |  |  | |  | |  | | | | |  | | |  | | | |
| Type of sport |  | Minutes/day | |  | | Days/month | | | | |  | | |  | | | |
| Type of sport |  | Minutes/day | |  | | Days/month | | | | |  | | |  | | | |
| Type of sport |  | Minutes/day | |  | | Days/month | | | | |  | | |  | | | |
| Climb stairs |  |  | |  | |  | | | | |  | | |  | | | |
| 3rd Dose |  | Floors/day | |  | | Days/month | | | | |  | | |  | | | |
| 1st Dose |  | Floors/day | |  | | Days/month | | | | |  | | |  | | | |
| In a typical week: |  |  | |  | |  | | | | |  | | |  | | | |
| How much time do you spend going shopping ON FOOT? |  |  | | 3rd Dose | | Min/week | | | | | 1st Dose | | | | Min/week | | |
| How much time do you spend cleaning the house? |  |  | | 3rd Dose | | Min/week | | | | | 1st Dose | | | | Min/week | | |
| How much time do you spend sitting on a workday? |  |  | | 3rd Dose | | Hours/day | | | | | 1st Dose | | | | Hours/day | | |

*Supplementary Table 1. Names and abbreviations of the inflammation biomarkers.*

| **Full name** | **Abbreviation** | **Full name** | **Abbreviation** |
| --- | --- | --- | --- |
| Adenosine Deaminase | ADA | Interleukin-15 receptor subunit alpha | IL-15RA |
| Artemin | ARTN | Interleukin-17A | IL-17A |
| Axin-1 | AXIN1 | Interleukin-17C | IL-17 |
| Beta-nerve growth factor | Beta-NGF | Interleukin-18 | IL-18 |
| Caspase-8 | CASP-8 | Interleukin-18 receptor 1 (IL-18R1) | IL-18R1 |
| C-C motif chemokine 3 | CCL3 | Interleukin-20 | IL-20 |
| C-C motif chemokine 4 | CCL4 | Interleukin-20 receptor subunit alpha | IL-20RA |
| C-C motif chemokine 19 | CCL19 | Interleukin-22 receptor subunit alpha-1 | IL-22 RA1 |
| C-C motif chemokine 20 | CCL20 | Interleukin-24 | IL-24 |
| C-C motif chemokine 23 | CCL23 | Interleukin-33 | IL-33 |
| C-C motif chemokine 25 | CCL25 | Latency-associated peptide transforming growth factor beta-1 | LAP TGF-beta-1 |
| C-C motif chemokine 28 | CCL28 | Leukemia inhibitory factor | LIF |
| CD40L receptor | CD40 | Leukemia inhibitory factor receptor | LIF-R |
| CUB domain-containing protein 1 | CDCP1 | Macrophage colony-stimulating factor 1 | CSF-1 |
| C-X-C motif chemokine 1 | CXCL1 | Matrix metalloproteinase-1 | MMP-1 |
| C-X-C motif chemokine 5 | CXCL5 | Matrix metalloproteinase-10 | MMP-10 |
| C-X-C motif chemokine 6 | CXCL6 | Monocyte chemotactic protein 1 | MCP-1 |
| C-X-C motif chemokine 9 | CXCL9 | Monocyte chemotactic protein 2 | MCP-2 |
| C-X-C motif chemokine 10 | CXCL10 | Monocyte chemotactic protein 3 | MCP-3 |
| C-X-C motif chemokine 11 | CXCL11 | Monocyte chemotactic protein 4 | MCP-4 |
| Cystatin D | CST5 | Natural killer cell receptor 2B4 | CD244 |
| Delta and Notch-like epidermal growth factor-related receptor | DNER | Neurotrophin-3 | NT-3 |
| Eotaxin | CCL11 | Neurturin | NRTN |
| Eukaryotic translation initiation factor 4E-binding protein 1 | 4E-BP1 | Oncostatin-M | OSM |
| Fibroblast growth factor 21 | FGF-21 | Osteoprotegerin | OPG |
| Fibroblast growth factor 23 | FGF-23 | Programmed cell death 1 ligand 1 | PD-L1 |
| Fibroblast growth factor 5 | FGF-5 | Protein S100-A12 | EN-RAGE |
| Fibroblast growth factor 19 | FGF-19 | Signaling lymphocytic activation molecule | SLAMF1 |
| Fms-related tyrosine kinase 3 ligand | Flt3L | SIR2-like protein 2 | SIRT2 |
| Fractalkine | CX3CL1 | STAM-binding protein | STAMBP |
| Glial cell line-derived neurotrophic factor | GDNF | Stem cell factor | SCF |
| Hepatocyte growth factor | HGF | Sulfotransferase 1A1 | ST1A1 |
| Interferon gamma | IFN-gamma | T cell surface glycoprotein CD6 isoform | CD6 |
| Interleukin-1 alpha | IL-1 alpha | T-cell surface glycoprotein CD5 | CD5 |
| Interleukin-2 | IL-2 | T-cell surface glycoprotein CD8 alpha chain | CD8A |
| Interleukin-2 receptor subunit beta | IL-2RB | Thymic stromal lymphopoietin | TSLP |
| Interleukin-4 | IL-4 | TNF-beta | TNFB |
| Interleukin-5 | IL5 | TNF-related activation-induced cytokine | TRANCE |
| Interleukin-6 | IL6 | TNF-related apoptosis-inducing ligand | TRAIL |
| Interleukin-7 | IL-7 | Transforming growth factor alpha | TGF-alpha |
| Interleukin-8 | IL-8 | Tumor necrosis factor (Ligand) superfamily, member 12 | TWEAK |
| Interleukin-10 | IL10 | Tumor necrosis factor | TNF |
| Interleukin-10 receptor subunit alpha | IL-10RA | Tumor necrosis factor ligand superfamily member 14 | TNFSF14 |
| Interleukin-10 receptor subunit beta | IL-10RB | Tumor necrosis factor receptor superfamily member 9 | TNFRSF9 |
| Interleukin-12 subunit beta | IL-12B | Urokinase-type plasminogen activator | uPA |
| Interleukin-13 | IL-13 | Vascular endothelial growth factor A | VEGF-A |

*Supplementary Table 2. Biological pathways mostly covered by the inflammation biomarkers.*

| Immune system | Signal transduction | Disease | Metabolism | Others |
| --- | --- | --- | --- | --- |
| CASP-8 | ARTN | ADA | AXIN1 | CD5 |
| CCL11 | AXIN1 | AXIN1 | CSF-1 | CD6 |
| CCL19 | CASP-8 | CASP-8 | 4E-BP1 | CDCP1 |
| CCL20 | CCL11 | FGF19 | FGF-23 | CST5 |
| CCL3 | CCL19 | FGF-23 | IFN-gamma | FGF-21 |
| CCL4 | CCL20 | FLT3LG | IL6 | MCP-2 |
| CD40 | CCL23 | IL10 | LIF-R | MMP-10 |
| CD8A | CCL25 | IL-17A | MMP-1 | SIRT2 |
| CSF-1 | CCL28 | IL-18 | STAMBP | SLAMF1 |
| CXCL1 | CCL3 | IL6 | TGF-alpha |  |
| CXCL10 | CCL4 | SCF | LAP TGF-beta-1 |  |
| EN-RAGE | CX3CL1 | TGF-alpha | VEGF-A |  |
| FLT3LG | CXCL1 | LAP TGF-beta-1 |  |  |
| HGF | CXCL10 | VEGF-A |  |  |
| IFN-gamma | CXCL11 |  |  |  |
| IL10 | CXCL5 |  |  |  |
| IL-10RA | CXCL6 |  |  |  |
| IL-10RB | CXCL9 |  |  |  |
| IL-12B | DNER |  |  |  |
| IL-13 | 4E-BP1 |  |  |  |
| IL-15RA | FGF19 |  |  |  |
| IL-17A | FGF-23 |  |  |  |
| IL-17C | FGF-5 |  |  |  |
| IL-18 | FLT3LG |  |  |  |
| IL-18R1 | GDNF |  |  |  |
| IL-20 | HGF |  |  |  |
| IL-20RA | IL-2RB |  |  |  |
| IL22RA1 | IL5 |  |  |  |
| IL-2RB | IL6 |  |  |  |
| IL-4 | IL-8 |  |  |  |
| IL5 | MCP-1 |  |  |  |
| IL6 | MCP-3 |  |  |  |
| IL-7 | MCP-4 |  |  |  |
| IL-8 | NRTN |  |  |  |
| LIF-R | NT-3 |  |  |  |
| TNFB | PD-L1 |  |  |  |
| MCP-1 | SCF |  |  |  |
| MMP-1 | TGF-alpha |  |  |  |
| OPG | LAP TGF-beta-1 |  |  |  |
| OSM | TNF |  |  |  |
| PD-L1 | TRAIL |  |  |  |
| LAP TGF-beta-1 | VEGF-A |  |  |  |
| TNF |  |  |  |  |
| LIF |  |  |  |  |
| TNFRSF9 |  |  |  |  |
| TNFSF14 |  |  |  |  |
| TRANCE |  |  |  |  |
| TSLP |  |  |  |  |
| TWEAK |  |  |  |  |
| uPA |  |  |  |  |
| VEGF-A |  |  |  |  |

*Supplementary Table 3. Mean, standard deviation (SD) and maximum and minimum values of the days that had elapsed between t0 and the following blood extractions.*

| Variable | Mean | SD | Min | Max |
| --- | --- | --- | --- | --- |
| days-t1 | 21.14 | 1.08 | 20 | 31 |
| days-t2 | 35.26 | 1.24 | 34 | 45 |
| days-t3 | 117.31 | 6.90 | 109 | 140 |
| days-t4 | 264.50 | 15.73 | 232 | 315 |
| days-t5 | 324.95 | 14.23 | 250 | 364 |

*Supplementary table 4. Effect of anti-COVID-19 vaccination in the levels of biomarkers.*

| Biomarker | t0 (median (IQR)) | t1(median (IQR)) | t2 (median (IQR)) | p-value | q-value |
| --- | --- | --- | --- | --- | --- |
| ADA | 5.00(4.57,5.35) | 5.07(4.75,5.50) | 4.89(4.55,5.19) | **<0.001** | **0.036** |
| ARTN | -0.45(-0.68,-0.10) | -0.49(-0.79,-0.14) | -0.47(-0.70,-0.27) | 0.674 | 0.913 |
| AXIN1 | 2.17(1.45,2.57) | 2.05(1.36,2.84) | 1.81(1.09,2.74) | 0.562 | 0.913 |
| CASP-8 | 6.03(5.54,6.49) | 5.46(4.89,5.96) | 5.59(5.15,6.17) | **<0.001** | **0.036** |
| CCL11 | 8.03(7.71,8.44) | 8.07(7.82,8.41) | 8.04(7.59,8.27) | 0.246 | 0.913 |
| CCL19 | 10.60(10.23,11.11) | 10.70(10.32,11.22) | 10.60(10.08,10.97) | 0.078 | 0.913 |
| CCL20 | 7.86(7.38,8.43) | 7.87(7.36,8.69) | 7.84(7.36,8.48) | 0.286 | 0.913 |
| CCL23 | 11.98(11.81,12.23) | 11.99(11.75,12.19) | 12.06(11.79,12.30) | 0.326 | 0.913 |
| CCL25 | 5.62(5.13,6.12) | 6.00(5.49,6.55) | 6.07(5.41,6.61) | **<0.001** | **0.036** |
| CCL28 | 1.46(1.17,1.70) | 1.36(1.18,1.59) | 1.40(1.11,1.76) | 0.181 | 0.913 |
| CCL3 | 7.41(6.78,8.08) | 7.33(6.71,8.15) | 7.27(6.76,8.37) | 0.286 | 0.913 |
| CCL4 | 7.77(7.39,8.07) | 7.67(7.27,8.17) | 7.54(7.15,7.99) | **0.004** | 0.268 |
| CD244 | 5.95(5.70,6.15) | 5.88(5.73,6.02) | 5.97(5.71,6.13) | **0.023** | 0.913 |
| CD40 | 12.05(11.81,12.29) | 12.02(11.77,12.19) | 11.99(11.75,12.21) | **0.012** | 0.696 |
| CD5 | 6.22(6.03,6.43) | 6.19(6.01,6.37) | 6.25(6.08,6.43) | **<0.001** | **0.036** |
| CD6 | 5.50(5.21,5.81) | 5.42(5.16,5.72) | 5.50(5.28,5.83) | **0.006** | 0.378 |
| CD8α | 8.66(8.23,9.08) | 8.81(8.29,9.30) | 8.60(7.87,9.14) | **<0.001** | **0.036** |
| CDCP1 | 3.74(3.45,4.18) | 3.72(3.37,4.13) | 3.84(3.48,4.06) | **0.018** | 0.913 |
| CSF1 | 10.18(10.04,10.29) | 10.17(10.05,10.27) | 10.17(10.04,10.27) | 0.119 | 0.913 |
| CST5 | 7.70(7.36,8.15) | 7.68(7.29,8.17) | 7.62(7.28,8.18) | 0.336 | 0.913 |
| CX3CL1 | 5.84(5.65,6.11) | 6.00(5.73,6.22) | 5.92(5.75,6.23) | 0.131 | 0.913 |
| CXCL1 | 10.22(9.97,10.56) | 10.21(9.93,10.53) | 10.23(10.00,10.53) | 0.450 | 0.913 |
| CXCL10 | 8.70(8.33,9.13) | 8.62(8.30,8.97) | 8.69(8.31,9.04) | **<0.001** | **0.036** |
| CXCL11 | 8.09(7.74,8.61) | 7.98(7.54,8.57) | 8.01(7.71,8.43) | **0.008** | 0.488 |
| CXCL5 | 12.97(12.55,13.21) | 12.90(12.53,13.18) | 12.89(12.46,13.21) | 0.913 | 0.913 |
| CXCL6 | 9.47(9.13,9.75) | 9.44(9.05,9.75) | 9.36(9.10,9.75) | **0.018** | 0.913 |
| CXCL9 | 7.29(6.85,7.68) | 7.16(6.82,7.56) | 7.28(6.93,7.80) | 0.307 | 0.913 |
| DNER | 9.81(9.66,9.94) | 9.76(9.61,9.91) | 9.75(9.65,9.90) | 0.181 | 0.913 |
| EBP1 | 2.41(1.76,2.96) | 2.38(1.67,3.01) | 1.87(1.47,2.51) | **0.005** | 0.320 |
| EN-RAGE | 7.60(6.84,8.30) | 7.27(6.49,7.90) | 7.22(6.46,7.79) | **<0.001** | **0.036** |
| FGF19 | 9.18(8.47,9.79) | 9.36(8.91,9.88) | 9.23(8.55,9.87) | **0.021** | 0.913 |
| FGF21 | 4.82(3.93,5.50) | 5.20(4.23,5.99) | 4.84(3.56,5.77) | **0.016** | 0.896 |
| FGF23 | 0.15(-0.18,0.34) | 0.32(-0.06,0.56) | 0.12(-0.17,0.40) | 0.083 | 0.913 |
| FGF5 | 1.09(0.90,1.37) | 1.11(0.91,1.34) | 1.06(0.73,1.24) | 0.063 | 0.913 |
| FLT3L | 10.11(9.85,10.35) | 10.12(9.75,10.28) | 10.11(9.80,10.38) | 0.105 | 0.913 |
| GDNF | 1.30(1.04,1.51) | 1.28(1.11,1.61) | 1.23(0.96,1.51) | **0.052** | 0.913 |
| HGH | 9.62(9.36,9.97) | 9.49(9.25,9.86) | 9.58(9.21,9.89) | **0.020** | 0.913 |
| IFNg | 7.89(7.39,8.42) | 7.88(7.35,8.27) | 7.94(7.47,8.42) | 0.428 | 0.913 |
| IL-10RA | 0.13(-0.18,0.98) | 0.10(-0.21,0.91) | 0.05(-0.20,0.88) | 0.148 | 0.913 |
| IL-10RB | 6.85(6.60,6.96) | 6.74(6.62,6.95) | 6.80(6.64,7.00) | **0.005** | 0.320 |
| IL-12B | 6.44(6.12,6.78) | 6.41(6.05,6.68) | 6.42(6.07,6.81) | 0.428 | 0.913 |
| IL-15RA | 1.38(1.17,1.56) | 1.41(1.19,1.58) | 1.38(1.16,1.64) | 0.825 | 0.913 |
| IL-17 | 0.56(0.27,0.93) | 0.55(0.23,0.89) | 0.52(0.17,0.82) | 0.138 | 0.913 |
| IL-17C | 1.69(1.36,2.21) | 1.90(1.58,2.53) | 1.61(1.31,2.19) | **<0.001** | **0.036** |
| IL-18R1 | 7.91(7.57,8.15) | 7.81(7.48,8.05) | 7.87(7.60,8.05) | **<0.001** | **0.036** |
| IL-20 | -0.07(-0.24,0.08) | -0.08(-0.28,0.00) | -0.13(-0.31,-0.01) | **0.015** | 0.855 |
| IL-20a | 0.25(0.00,0.59) | 0.30(-0.02,0.64) | 0.25(0.00,0.62) | 0.674 | 0.913 |
| IL-2RB | 0.83(0.58,1.11) | 0.80(0.53,1.00) | 0.87(0.60,1.12) | 0.207 | 0.913 |
| IL-8 | 7.86(7.19,8.60) | 7.63(7.17,8.31) | 7.72(7.29,8.95) | 0.103 | 0.913 |
| IL-10 | 2.57(2.21,2.90) | 2.64(2.32,3.00) | 2.62(2.22,2.99) | 0.286 | 0.913 |
| IL-18 | 9.67(9.32,10.07) | 9.69(9.34,9.96) | 9.75(9.51,10.01) | 0.399 | 0.913 |
| IL-6 | 2.80(2.46,3.48) | 2.71(2.23,3.33) | 2.81(2.42,3.54) | 0.731 | 0.913 |
| IL-7 | 2.77(2.32,3.02) | 2.72(2.41,3.08) | 2.85(2.51,3.15) | **0.035** | 0.913 |
| LIF | -0.68(-0.82,-0.46) | -0.74(-0.91,-0.52) | -0.66(-0.86,-0.48) | 0.478 | 0.913 |
| LIFR | 4.23(4.10,4.43) | 4.24(4.06,4.37) | 4.31(4.11,4.45) | **0.012** | 0.696 |
| MCP-1 | 12.81(12.48,13.18) | 12.80(12.50,13.14) | 12.81(12.44,13.09) | 0.083 | 0.913 |
| MCP-2 | 10.02(9.63,10.52) | 10.05(9.54,10.39) | 9.92(9.59,10.30) | **0.001** | 0.071 |
| MCP-3 | 2.67(2.24,3.04) | 2.63(2.27,3.21) | 2.74(2.35,3.10) | 0.688 | 0.913 |
| MCP-4 | 16.25(15.83,16.59) | 16.2(15.68,16.51) | 16.13(15.63,16.43) | **0.011** | 0.660 |
| MMP1 | 16.14(15.53,16.42) | 16.05(15.53,16.46) | 16.12(15.53,16.50) | 0.085 | 0.913 |
| MMP10 | 9.07(8.79,9.50) | 9.15(8.78,9.57) | 9.02(8.50,9.44) | **0.046** | 0.913 |
| NRTN | -0.50(-0.66,-0.22) | -0.49(-0.67,-0.30) | -0.55(-0.76,-0.33) | 0.078 | 0.913 |
| NT3 | 2.49(2.19,2.81) | 2.66(2.40,2.90) | 2.49(1.99,2.76) | **0.026** | 0.913 |
| OPG | 10.31(10.12,10.54) | 10.28(10.10,10.52) | 10.30(10.16,10.57) | **0.003** | 0.204 |
| OSM | 7.35(6.83,7.81) | 7.01(6.56,7.74) | 7.14(6.61,7.74) | 0.063 | 0.913 |
| PDL1 | 6.49(6.26,6.65) | 6.41(6.25,6.62) | 6.46(6.32,6.63) | **0.026** | 0.913 |
| SCF | 9.89(9.69,10.08) | 9.90(9.71,10.05) | 9.86(9.64,10.08) | 0.272 | 0.913 |
| SIRT2 | 2.21(1.70,2.62) | 1.98(1.48,2.56) | 1.93(1.45,2.44) | **0.046** | 0.913 |
| SLAMF1 | 2.08(1.88,2.30) | 2.12(1.85,2.29) | 2.12(1.91,2.35) | 0.307 | 0.913 |
| ST1A1 | 4.01(3.43,4.82) | 4.29(3.65,4.93) | 3.75(2.89,4.54) | **0.021** | 0.913 |
| STAMBP | 4.79(4.46,5.12) | 4.67(4.37,4.97) | 4.64(4.38,5.00) | **0.002** | 0.138 |
| TGF_A | 5.63(5.25,6.09) | 5.51(5.05,5.87) | 5.46(4.95,5.96) | **0.023** | 0.913 |
| TGFB1 | 8.07(7.74,8.34) | 8.05(7.76,8.34) | 8.00(7.73,8.23) | 0.218 | 0.913 |
| TNF | 2.27(1.94,2.81) | 2.43(2.04,3.13) | 2.16(1.63,2.78) | **0.002** | 0.138 |
| TNFB | 5.51(5.25,5.90) | 5.53(5.22,5.76) | 5.52(5.22,5.92) | **<0.001** | **0.036** |
| TNFRSF9 | 6.29(6.05,6.52) | 6.28(6.10,6.54) | 6.39(6.18,6.57) | **0.029** | 0.913 |
| TNFSF14 | 7.70(7.17,8.22) | 7.47(6.93,8.09) | 7.51(6.80,8.04) | 0.029 | 0.913 |
| TRAIL | 8.91(8.68,9.07) | 8.97(8.80,9.14) | 8.84(8.61,9.03) | **0.005** | 0.320 |
| TRANCE | 5.57(5.16,5.94) | 5.59(5.28,6.04) | 5.60(5.35,5.95) | 0.277 | 0.913 |
| TWEAK | 10.07(9.90,10.28) | 10.09(9.86,10.29) | 10.06(9.85,10.30) | 0.648 | 0.913 |
| UPA | 10.92(10.72,11.12) | 10.86(10.68,11.04) | 10.94(10.75,11.17) | **<0.001** | **0.036** |
| VEGF_A | 12.66(12.22,13.06) | 12.59(12.16,13.00) | 12.70(12.23,13.03) | **0.007** | 0.434 |

*p-value corresponds to the Friedman’s test. q-value corresponds to the Benhamini-Hochber correction for multiple comparisons. Statistically significant (<0.05) p and q values are marked in bold. IQR: interquartile range (percentile 25-percentile 75).*

*Supplementary Table 5. Principal component analysis results – group ‘’Others’’.*

| variable | PC1 | PC2 | Unexplained |
| --- | --- | --- | --- |
| CD5 | 0.4527 | -0.3120 | 0.3016 |
| CD6 | 0.3960 | -0.3405 | 0.4135 |
| CDCP1 | 0.3948 | 0.2883 | 0.4574 |
| MMP-10 | 0.3831 | 0.0930 | 0.5763 |
| MCP-2 | 0.3102 | 0.3932 | 0.5354 |
| FGF-21 | 0.2586 | 0.6027 | 0.3564 |
| CST5 | 0.2430 | -0.0705 | 0.8276 |
| SIRT2 | 0.2388 | -0.3649 | 0.6727 |
| SLAMF1 | 0.2377 | -0.1975 | 0.7921 |
| KMO total | 0.6988 |  |  |

Eigen vector values for the PC1 and PC2 components are shown in the table, as well as the Kaiser-Meyer-Olkin (KMO) measure of sampling adequacy.

*Supplementary Table 6. Principal component analysis results – group ‘’Signal Transduction’’.*

| variable | PC1 | PC2 | Unexplained |
| --- | --- | --- | --- |
| MCP-4 | 0.2375 | 0.1015 | 0.4399 |
| MCP-1 | 0.2214 | 0.1717 | 0.4374 |
| CXCL11 | 0.2210 | 0.0293 | 0.5448 |
| CXCL1 | 0.2209 | -0.0318 | 0.5448 |
| HGF | 0.2061 | -0.1838 | 0.4821 |
| CCL4 | 0.2049 | -0.2455 | 0.3887 |
| CXCL6 | 0.2006 | 0.0453 | 0.6200 |
| CASP-8 | 0.2004 | -0.1591 | 0.5347 |
| IL-8 | 0.1998 | -0.2803 | 0.3404 |
| TRAIL | 0.1965 | 0.1699 | 0.5360 |
| CCL11 | 0.1948 | 0.2073 | 0.4898 |
| AXIN1 | 0.1922 | -0.1364 | 0.5893 |
| CCL3 | 0.1913 | -0.3276 | 0.2648 |
| MCP-3 | 0.1786 | -0.0348 | 0.7003 |
| VEGF-A | 0.1756 | 0.0335 | 0.7104 |
| CXCL5 | 0.1675 | -0.0696 | 0.7223 |
| DNER | 0.1622 | 0.1665 | 0.6540 |
| CXCL10 | 0.1586 | 0.1486 | 0.6855 |
| SCF | 0.1534 | -0.0584 | 0.7696 |
| FGF-5 | 0.1519 | 0.0855 | 0.7594 |
| CCL28 | 0.1484 | 0.0294 | 0.7930 |
| PD-L1 | 0.1465 | 0.1915 | 0.6658 |
| TNF | 0.1432 | -0.2443 | 0.5898 |
| CCL23 | 0.1406 | 0.1244 | 0.7598 |
| TGF-alpha | 0.1393 | -0.2456 | 0.5976 |
| FLT3LG | 0.1392 | 0.1885 | 0.6892 |
| CXCL9 | 0.1285 | 0.1703 | 0.7401 |
| IL6 | 0.1275 | -0.2060 | 0.6927 |
| CCL19 | 0.1190 | -0.0424 | 0.8622 |
| 4E-BP1 | 0.1051 | 0.1026 | 0.8589 |
| FGF-23 | 0.1043 | 0.0865 | 0.8717 |
| CCL20 | 0.1035 | -0.062 | 0.8867 |
| NT-3 | 0.0986 | 0.0935 | 0.8777 |
| GDNF | 0.0921 | 0.1488 | 0.8396 |
| FGF19 | 0.0910 | 0.1213 | 0.8690 |
| CX3CL1 | 0.0716 | 0.2322 | 0.7534 |
| NRTN | 0.0703 | -0.1005 | 0.9169 |
| IL-2RB | 0.0663 | 0.0469 | 0.9511 |
| LAP TGF-beta-1 | 0.0630 | 0.0616 | 0.9492 |
| CCL25 | 0.0606 | 0.2706 | 0.6956 |
| IL5 | 0.0401 | 0.0142 | 0.9844 |
| ARTN | 0.0305 | -0.0170 | 0.9903 |
| KMO Total | 0.7056 |  |  |

Eigen vector values for the PC1 and PC2 components are shown in the table, as well as the Kaiser-Meyer-Olkin (KMO) measure of sampling adequacy .

*Supplementary Table 7. Principal component analysis results – group ‘Immune system’’.*

| variable | PC1 | PC2 | Unexplained |
| --- | --- | --- | --- |
| CD40 | 0.2295 | -0.0121 | 0.4472 |
| TNFSF14 | 0.2205 | -0.2065 | 0.3015 |
| HGF | 0.2189 | -0.1468 | 0.4023 |
| CASP-8 | 0.1987 | -0.1562 | 0.4781 |
| OSM | 0.1986 | -0.2479 | 0.3143 |
| uPA | 0.1964 | 0.0884 | 0.5609 |
| MCP-1 | 0.1950 | 0.0813 | 0.5721 |
| OPG | 0.1912 | 0.1017 | 0.5709 |
| IL-10RB | 0.1850 | 0.2230 | 0.4208 |
| CXCL1 | 0.1841 | -0.0602 | 0.6285 |
| VEGF-A | 0.1801 | 0.0297 | 0.6558 |
| CXCL10 | 0.1796 | 0.1560 | 0.5539 |
| TWEAK | 0.1747 | 0.1349 | 0.5993 |
| CCL4 | 0.1733 | -0.2634 | 0.3779 |
| EN-RAGE | 0.1708 | -0.1721 | 0.5629 |
| PD-L1 | 0.1690 | 0.2128 | 0.5003 |
| IL-8 | 0.1608 | -0.3005 | 0.3294 |
| TNFRSF9 | 0.1587 | 0.1540 | 0.6308 |
| CCL3 | 0.1559 | -0.3310 | 0.2601 |
| IL-18 | 0.1549 | 0.1821 | 0.6016 |
| IL-18R1 | 0.1527 | 0.0995 | 0.7116 |
| CSF-1 | 0.1499 | 0.1604 | 0.6504 |
| LIF-R | 0.1468 | 0.1848 | 0.6229 |
| IL-12B | 0.1467 | 0.0624 | 0.7571 |
| FLT3LG | 0.1405 | 0.1432 | 0.7023 |
| CCL11 | 0.1379 | 0.0629 | 0.7830 |
| IL6 | 0.1351 | -0.1483 | 0.7114 |
| IL-15RA | 0.1296 | 0.1709 | 0.6946 |
| CCL20 | 0.1217 | -0.0267 | 0.8416 |
| IL-17A | 0.1150 | 0.0297 | 0.8575 |
| CCL19 | 0.1105 | -0.0374 | 0.8658 |
| LAP TGF-beta-1 | 0.1033 | -0.1138 | 0.8307 |
| IL-2RB | 0.1016 | 0.1030 | 0.8448 |
| TNF | 0.0993 | -0.2837 | 0.5405 |
| IL22RA1 | 0.0975 | 0.0655 | 0.8813 |
| TRANCE | 0.0968 | 0.0812 | 0.8725 |
| TNFB | 0.0945 | 0.0941 | 0.8671 |
| TSLP | 0.0896 | -0.0447 | 0.9070 |
| IL-17C | 0.0892 | -0.0395 | 0.9098 |
| IL-20RA | 0.0841 | 0.0313 | 0.9214 |
| MMP-1 | 0.0817 | 0.0524 | 0.9178 |
| IL10 | 0.0602 | -0.0776 | 0.9354 |
| IL-4 | 0.0498 | 0.0253 | 0.9711 |
| IL5 | 0.0496 | 0.0346 | 0.9688 |
| IFN-gamma | 0.0479 | 0.1670 | 0.8524 |
| IL-20 | 0.0458 | 0.0028 | 0.9780 |
| IL-7 | 0.0300 | 0.0601 | 0.9745 |
| IL-13 | 0.0273 | 0.0462 | 0.9828 |
| IL-10RA | 0.0260 | 0.0083 | 0.9926 |
| LIF | -0.0102 | -0.0206 | 0.9970 |
| CD8A | -0.0404 | -0.0444 | 0.9742 |
| KMO Total | 0.6607 |  |  |

Eigen vector values for the PC1 and PC2 components are shown in the table, as well as the Kaiser-Meyer-Olkin (KMO) measure of sampling adequacy .

*Supplementary Table 8. Principal component analysis results – group ‘Metabolism and protein expression’’.*

| variable | PC1 | PC2 | Unexplained |
| --- | --- | --- | --- |
| STAMBP | 0.3973 | 0.0877 | 0.5031 |
| AXIN1 | 0.3804 | -0.4040 | 0.3050 |
| VEGF-A | 0.3383 | 0.3316 | 0.4796 |
| LIF-R | 0.3054 | 0.0931 | 0.7000 |
| CSF-1 | 0.3004 | 0.4105 | 0.4643 |
| TGF-alpha | 0.2946 | 0.0745 | 0.7247 |
| LAP TGF-beta-1 | 0.2940 | -0.3851 | 0.5070 |
| FGF-23 | 0.2862 | -0.1894 | 0.6933 |
| 4E-BP1 | 0.2789 | -0.3800 | 0.5395 |
| MMP-1 | 0.1840 | 0.1616 | 0.8560 |
| IL6 | 0.1668 | 0.3743 | 0.6997 |
| IFN-gamma | 0.0696 | 0.2040 | 0.9213 |
| KMO Total | 0.6346 |  |  |

Eigen vector values for the PC1 and PC2 components are shown in the table, as well as the Kaiser-Meyer-Olkin (KMO) measure of sampling adequacy .

*Supplementary Table 9. Principal component analysis results – group ‘Disease’.*

| variable | PC1 | PC2 | Unexplained |
| --- | --- | --- | --- |
| CASP-8 | 0.4198 | -0.0486 | 0.4702 |
| VEGF-A | 0.3931 | -0.2128 | 0.4533 |
| AXIN1 | 0.3659 | 0.3624 | 0.3515 |
| TGF-alpha | 0.3217 | 0.0653 | 0.6834 |
| LAP TGF-beta-1 | 0.2792 | 0.3969 | 0.4683 |
| ADA | 0.2620 | -0.2964 | 0.6284 |
| IL6 | 0.2438 | -0.1730 | 0.7661 |
| FGF-23 | 0.2419 | 0.3084 | 0.6448 |
| FLT3LG | 0.2131 | -0.2226 | 0.7705 |
| IL-17A | 0.2066 | -0.2195 | 0.7813 |
| IL-18 | 0.1872 | -0.3895 | 0.6073 |
| FGF19 | 0.1341 | 0.1369 | 0.9108 |
| IL10 | 0.1252 | -0.0822 | 0.9405 |
| SCF | 0.1025 | 0.4140 | 0.6430 |
| KMO Total | 0.6126 |  |  |

Eigen vector values for the PC1 and PC2 components are shown in the table, as well as the Kaiser-Meyer-Olkin (KMO) measure of sampling adequacy.
